# Efficient steganalysis using convolutional auto encoder network to ensure original image quality

**DOI:** 10.7717/peerj-cs.356

**Published:** 2021-02-16

**Authors:** Mallikarjuna Reddy Ayaluri, Sudheer Reddy K., Srinivasa Reddy Konda, Sudharshan Reddy Chidirala

**Affiliations:** 1 Computer Science and Engineering, Anurag University, Hyderabad, India; 2 Information Technology, Anurag University, Hyderabad, India; 3 Computer Science and Engineering, BVRIT Hyderabad College of Engineering for Women, Hyderabad, India; 4 Computer Science and Engineering, GNITS, Hyderabad, India

**Keywords:** Steganalysis, Deep neural network, Auto encoder, Non Gaussian noise, Image quality, Error cost, Convolutional auto encoder deep learning framework

## Abstract

Steganalysis is the process of analyzing and predicting the presence of hidden information in images. Steganalysis would be most useful to predict whether the received images contain useful information. However, it is more difficult to predict the hidden information in images which is computationally difficult. In the existing research method, this is resolved by introducing the deep learning approach which attempts to perform steganalysis tasks in effectively. However, this research method does not concentrate the noises present in the images. It might increase the computational overhead where the error cost adjustment would require more iteration. This is resolved in the proposed research technique by introducing the novel research method called Non-Gaussian Noise Aware Auto Encoder Convolutional Neural Network (NGN-AEDNN). Classification technique provides a more flexible way for steganalysis where the multiple features present in the environment would lead to an inaccurate prediction rate. Here, learning accuracy is improved by introducing noise removal techniques before performing a learning task. Non-Gaussian Noise Removal technique is utilized to remove the noises before learning. Also, Gaussian noise removal is applied at every iteration of the neural network to adjust the error rate without the involvement of noisy features. This proposed work can ensure efficient steganalysis by accurate learning task. Matlab has been employed to implement the method by performing simulations from which it is proved that the proposed research technique NGN-AEDNN can ensure the efficient steganalysis outcome with the reduced computational overhead when compared with the existing methods.

## Introduction

Multimedia data hiding methods play an important part in the telemedicine fields due to the presence of more sensitive information present on multimedia contents. They have been proposed to include delicate watermarks media like pictures or pdf archives, to ensure the validness of the material: any endeavor of adjustment of the help will change the watermark, demonstrating by doing as such the alteration. Data concealing systems can be utilized to advance the media, for example by including individual and restorative data inside medicinal pictures (Nilsson, 2014). Doing as such may prevent two noteworthy dangers in telemedicine. Right off the bat, these individual and restorative information end up out of reach to any unapproved person that approaches the electronic medicinal record, accordingly guaranteeing information privacy (Kim, 2015; Bengio, Courville & Vincent, 2013). Also, it prevents the hazard of unexpectedly blending the substance of wellbeing records between patients, as the most delicate data are embedded inside the most essential medias. Different uses of steganography (calculations that shroud data inside a host mixed media bolster) and steganalysis (the opposite, that is, instruments that recognize the nearness of covered up, informal data) are now and again detailed, making this data security field of research a promising strategy for telemedicine (Böhmer et al., 2013; Larose, 2014). Steganalysis is used for finding whether an article contains (or not) a shrouded message. This work centers around picture steganalysis, that is, when cover objects are pictures. It happens in the setting where the picture space is known. Extra learning on the installing calculations and the payload can help the steganalyzer apparatus.

A standout amongst the most prominent procedures accessible in information mining to perform steganalysis is machine learning. Machine learning is giving an adaptable method to analysts to examine and foresee the structure of databases naturally by anticipating the essential highlights from the crude information (Radford, Metz & Chintala, 2015). It enables analysts to learn both component nearness and foreseeing the arrangement dependent on those extricated highlights to give the required yield. Here element learning is the intricate assignment that can be improved by machine learning strategies. For instance, arrangement methods can be utilized to highlight realizing which will process and change over the highlights computationally adaptable way (Pathak et al., 2016). Every sort of picture would comprise of explicit kind of highlights which is increasingly hard to foresee and break down algorithmically (Zenil, Kiani & Tegnér, 2017). Hence, it is required to present the more powerful systems which can uncover the structure of highlight nearness in the medicinal imaging and foresee the analysis result all the more ideally without depending on the outside techniques.

At present deep architectures like deep belief networks (DBN) plays a more important role in prediction system s and unsupervised feature learning. More research has been studied and widely applied by using DBN and stacked autoencoders (SAE) for the feature learning and prediction process (Radford, Metz & Chintala, 2015). It is more popular among researchers by successfully learning the feature values and providing a more successful outcome. Deep architecture is a more powerful technique in nature to discover the more useful information from the database by recognizing and diagnosing the outcome more accurately even in the case of deep architectures. It can extract more useful information from the database without worrying about the labeled information is how deep it is. It also provides a more flexible and accurate outcome (Eigen et al., 2013).

Deep architecture is found to be the more popular technique in the real-world; still, it finds many challenges and problems with the detection of noises and outliers present in the database with real-world data such as medical imaging data features (Qi et al., 2014). Thus it is required to concentrate on the analysis of database features and it needs to be found in more detail by predicting the noises and outliers (Gopalan, Li & Chellappa, 2014). It is required to take more effort to handle the scenarios with the presence of noises in the database. This is focused on the proposed research technique by introducing the novel research method called Non-Gaussian Noise Aware Auto Encoder Deep Neural Network (NGN-AEDNN). Classification technique provides a more flexible way for steganalysis where the multiple features present in the environment would lead to an inaccurate prediction rate.

The overall organization of the research method is given as follows: In this section detailed introduction about the deep neural network and their needs and roles has been given. In “Related Works”, varying related research methodologies that attempt to perform deep neural learning with increased prediction accuracy have been given. In “Non-Gaussian Noise Aware Steganalysis Process”, the proposed research methodology has been discussed in detail along with suitable examples and explanations. In “Results and Discussion”, the experimental evaluation of the proposed research technique has been shown by comparing it with different research techniques. In “Conclusions”, the overall conclusion of the proposed research methodology has been given in terms of various performance metrics related to accuracy evaluation.

## Related Works

In Schmidhuber (2015), an overview of deep learning in the neural network is discussed in detail. This work provides an application that adapts the deep learning procedure for accomplishing the information discovery accurately. This provides a clear description of the interlinks and the shortage path between the different nodes. In Deng (2014), the authors discussed deep neural network-based learning in detail. This research method provides a clear view of architectures, algorithms, and applications of deep neural networking with suitable examples and explanations.

In Kwon et al. (2017), the authors introduced the deep learning architecture base on the restricted Boltzmann machine concept. The authors have analyzed the working status of deep neural networks in the network anomaly detection application from which it is found that the deep neural network can effectively operate on the anomaly detection scenarios. In Bhatia & Rana (2015), analysis evaluation is done by the authors to find the usefulness of deep learning in the real world under various scenarios. He evaluated the usefulness and effectiveness of DBN by comparing it with the Convolutional neural network (CNN) under different conditions on different applications.

In Lv et al. (2015), the authors performed a traffic flow prediction task by using the deep neural network. Here the traffic flow prediction is performed with the consideration of both spatial and temporal correlations on database features. The authors adapted the stacked autoencoder mode to accurately find the traffic flow prediction in an accurate manner with more iteration based on the greedy layer concept. In Ioannidou et al. (2017), the author’s performance survey analysis deep learning techniques over various applications with 3D data. Authors tempt to find the procedure of deep learning that ensures a better classification outcome. After analysis of this scenario under various conditions, it is found that the deep learning method can perform better with 3D data by placing it in 2D view and process them in the multiple layers and augmentation outcome.

In Romero, Gatta & Camps-Valls (2016), the authors introduced the single layer and deep convolutional networks based on a prediction system for the remote sensing data analysis. Here authors introduced the supervised deep convolutional network which can better operate on multi and hyper spectral imagery fields. Here authors concluded that the proposed research method cannot produce the optimal outcome with the presence of a high dimensional dataset with less labeled information. In Stober et al. (2015), the authors performed the analysis of various learning techniques with discriminative features which are measured from the Electroencephalography (EEG) recordings. Here Deep learning strategies are applied to data to analyze their performance. It is found the EEG cannot provide a better outcome with the presence of various discriminative features presence. A deep neural network can effectively analyze and predict the outcome even in case of the presence of more varying dimensional features.

In Zhong et al. (2016), Mallikarjuna Reddy et al. (2019), Mallikarjuna & Karuna Sree (2019), Chandrasekhara Reddy et al. (2019), Sudheer Reddy & Srinagesh (2013) and Sudheer Reddy, Varma & Reddy (2012), varying data learning techniques has been investigated under different scenarios. These techniques adapt to the network environment with different feature learning methods under varying models. Here data from various online repositories has been analyzed and processed in detail.

## Non-Gaussian Noise Aware Steganalysis Process

Classification technique provides a more flexible way for steganalysis where the multiple features present in the environment would lead to an inaccurate prediction rate. Here, learning accuracy is improved by introducing noise removal techniques before performing the learning task. Non-Gaussian Noise Removal technique is utilized to remove the noises before learning. And also Gaussian noise removal is applied at every iteration of the neural network to adjust the error rate without the involvement of noisy features. The proposed research method has been implemented in the Matlab environment to make it to adapt the large volume of images using the Hadoop Image Processing Interface (HIPI) that is an image processing public collection designed to be used with the Apache Hadoop MapReduce parallel programing framework. HIPI runs on a cluster that uses MapReduce algorithms to process high-throughput images. HIPI is a solution provider to archive a large collection of images on the Hadoop Distributed File System (HDFS) and make them ready for processing.

**Figure 1:**
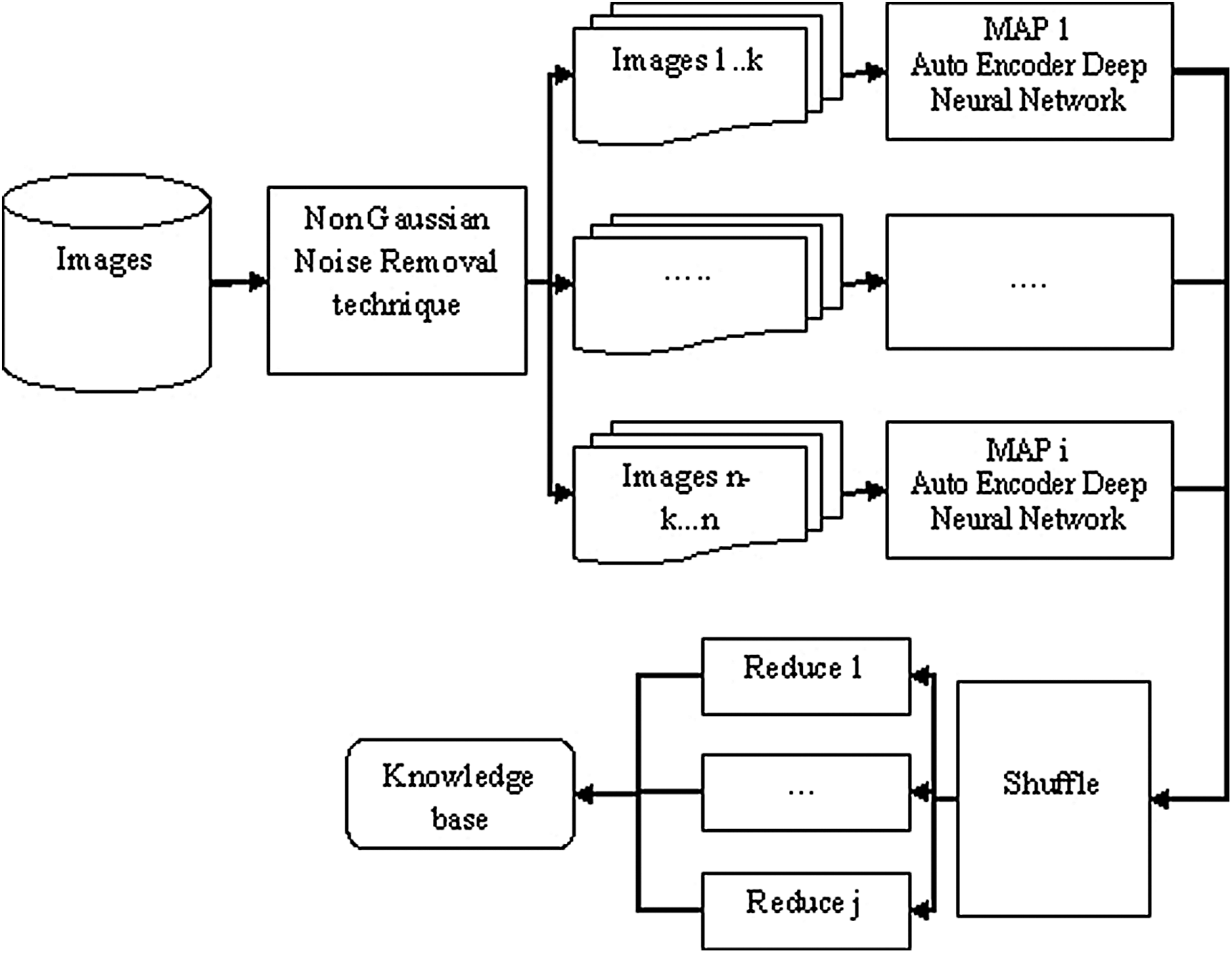
Processing flow of proposed work.

Figure 1, depicts the processing flow of the proposed research methodology when it is applied to the Hadoop environment. This Fig. 1 only depicts the training process of the learning module whose resultant depicts the knowledge base which indicates the learned feature knowledge. This simulation environment is implemented successfully and the testing phase is in process.

### Non-Gaussian noise aware auto encoder deep neural network

Steganalysis would become a more difficult task in case of the presence of Gaussian noise present on the images which will occur due to poor illumination. These Gaussian noise needs to be avoided first to ensure the accurate steganalysis outcome. Gaussian noise is a noise whose probability density function is equal to the normal pixel of images. It is required to predict the difference between the normal pixel value and Gaussian noised pixel value which is more difficult due to their similarity.

In this research method, Gaussian noise detection is done by using the correntropy measure. Correntropy measure is used to calculate the similarity between the distributions of pixels. In this research method, measurement of correntropy is integrated with the autoencoder based deep neural network. This integrated mechanism namely Gaussian noise-aware autoencoder (GNAE) can ensure accurate and reliable steganalysis even in presence of Gaussian noise present on the images. This GNAE is combined with the deep neural network to ensure the accurate prediction of the Gaussian noises present on the images.

An Auto encoder is used to learn an efficient data coding in an unsupervised approach, When input layer transfer data to hidden layer, its weight multiply the incoming data. The input layer that passes the data through the activation function. The activation function uses filtering the noise using encoder techniques (for reference, digital filtering technique which removes noise, image smoothening and others).

From the hidden layer, the data will be transferred to the output layer through the decoder. Once the reconstruction takes place, it will create an image which is similar to that of the original input image after noise reduction. This is possible by removing extra bits which are added for encoding the input data.

In the following sub-sections, the correntropy measurement procedure and its integrated form with the autoencoder to perform steganalysis in an accurate manner is discussed in detail with suitable examples.

### Correntropy measurement procedure

Correntropy measure is used to calculate the similarity between the nearest pixels based on their distribution. Correntropy measure value between the two pixels namely *A* and *B* is calculated as like as follows:

(1) $${\rm{Correntrop}}{{\rm{y}}_\sigma }(A,B) = E[{k_\sigma }(A - B)]$$

where *E*[.] → Mathematical expectation

*k*_σ_ → normalized Gaussian kernel functions

σ → Kernel size

Correntropy measure is more or less similar to the Renyi quadratic entropy (Zhong et al., 2016) which is used to detect the similarities between the distributed data and ensure the detection and elimination of Gaussian noises present on images. The kernel function given in Eq. (1) is calculated as like as follows given in Eq. (2):

(2) $${{k}_{\rm{\sigma}}}(.) = \displaystyle{1 \over {\sqrt {2{\rm{\pi}{\rm{\sigma}}}} }}{\rm exp}\left( {\displaystyle{{{{(.)}^2}} \over {2{{\rm{\sigma}}^2}}}} \right)$$

From this equation, it can be clearly said that the correntropy measure is a positive and bounded value. The parameter σ in the equation depicts the correlation adjustment factor. Σ and high order moments are directionally proportional to each other where the increased σ value would also fasten the high order moments. Thus the equivalent distance would change from 2 norms to zero norm if the distance between the variables *A* and *B* gets larger. Thus it can detect the anomaly and irrelevant data from the database very accurately. The calculation procedure of correlation with the absence of knowledge about joint pdf between *A* and *B* with limited sample availability such as $\left\{ {\left( {{{a}_{i}},{{b}_{i}}} \right)} \right\}_{{t} = 1}^{N}$ of variables *A* and *B* are given in the following Eq. (3):

(3) $${{\widehat{{\rm Correntropy}}}_{\rm{\sigma}}}\left( {{A},{B}} \right) = \displaystyle{1 \over {N}}\mathop \sum \limits_{{t} = 1}^{N} {{k}_{\rm{\sigma} }}\left( {{{a}_{t}} - {{b}_{t}}} \right)$$

The above-mentioned Eqs. (1)–(3) used to predict the correntropy between the two single-pixel values which cannot be applied of the vector of pixel data. The calculation procedure of correntropy between two-pixel vectors *P* = (*p*_1_, …, *p_N_*)*^T^* and *Q* = (*q*_1_, …, *q_N_*)*^T^* is depicted in the following Eq. (4):

(4) $${\rm CIM}\left( {{P},{Q}} \right) = {\left( {{g}(0) - \displaystyle{1 \over {N}}\mathop \sum \limits_{{t} = 1}^{N} {g}\left( {{{e}_{i}}} \right)} \right)^{1/2}} = {\left( {{g}(0) - \displaystyle{1 \over {N}}\mathop \sum \limits_{{t} = 1}^{N} {g}({{{p}_{i}} - {{q}_{i}}})} \right)^{1/2}}$$

where *e_i_* → error which is calculated as given in Eq. (5) *g*(*x*) → Gaussian kernel which is calculated as like given in Eq. (6)

(5) $${e_i} = {p_i} - {q_i}$$

(6) $$g(x)\matrix{
   {\rm{\Delta }}  \cr 
    =   \cr 
 } {\mathop{\rm exp}\nolimits} \left( { - {{{x^2}} \over {2{\sigma ^2}}}} \right)$$

Here the maximum correntropy measure values of error *e_j_* is calculated as like as in Eq. (7):

(7) $$\max \displaystyle{1 \over {N}}\mathop \sum \limits_{{t} = 1}^{N} {g}\left( {{{e}_{i}}} \right)$$

The main goal of the autoencoders is to learn the features with a reduced reconstruction cost function. The proposed method Gaussian noise aware autoencoder (GNAE) is a three-layer network which includes the encoder and the decoder. The network structure of the proposed GNAE consists of one input layer with d inputs, one hidden layer, one reconstruction layer, and one activation function. GNAE is main concern is to remove the Gaussian noise presence from the input dataset, thus accurate learning can be ensured.

The encoder from the GNAE will transfer the input vector *a* ∈ *R^d^* to the hidden layer where the latent activity would be generated which is depicted as *b* ∈ *R^h^*. This latent activity value *b* will then transfer by a decoder to the output layer where the input reconstruction would be performed. The output derived from the reconstruction process in the output layer is depicted as *c* ∈ *R^d^*. The mathematical calculation procedure of these equations is depicted in the following Eqs. (8) and (9).

(8) $${b} = f(W_{b}{a} + q_b)$$

(9) $$c = f(W_z{b} + q_c)$$

where *W_b_* → Input to hidden layer weights

*W_z_* → hidden to output layer weights

*q_h_* → bias of hidden layer

*q_c_* → bias of output layer

*f*(*) → activation function

Here the activation function *f*(*) is taken as sigmoidal function for both encoder and decoder. The main objective of the proposed research method is depicted as follows:

(10) $$W_b = E_c^t = W$$

In Eq. (10), the values of the weights of GNAE method have been given. The deep learning architecture parameters are depicted as θ = {*W*, *q_b_*, *q_c_*} which is used to reconstruct the input data values from the output data values with a reduced reconstruction cost function. The reconstruction cost of GNAE is calculated as like given in Eq. (11) with the concern of mean square error and cross-entropy values between the input vector and output vector values.

(11) $$J_{\rm cost}({\rm{\theta}}) = L(a,c) + {\rm{\lambda}}\|J_{f}(a)\|_{F}^2$$

where λ → positive hyper parameter used to control the regularization parameter values

*J*(cost) → correntropy cost function

The reconstruction cost function is defined as:

(12) $$L(a,c) = \displaystyle{1 \over {m}}\mathop \sum \limits_{{t} = 1}^{m} \mathop \sum \limits_{{k} = 1}^{n} {{k}_{\rm{\sigma}}}(a_{tk} - c_{tk})$$

where *m* → number of input training samples

*n* → length of training samples

To support the robustness of the feature learning process, in this research method Jacobian norm mapping is considered *J_f_* (*a*) which is a nonlinear mapping value of encoding function *f*. This is used to map the hidden representation which is represented as *h* = *f*(*x*) ∈ *R^dh^*, This is calculated by taking the summation of extracted features from the images which are calculated as like given in Eq. (13):

(13) $$\|{J_f}(a)\|_{F}^2 = \mathop \sum \limits_{{t} = 1}^{{{d}_{h}}} \mathop \sum \limits_{{j} = 1}^{{{d}_{x}}} {\left( {\displaystyle{{{\rm{\vartheta}} {{h}_{i}}} \over {{\rm{\vartheta}} {{x}_{j}}}}} \right)^2} = \mathop \sum \limits_{{t} = 1}^{{{d}_{h}}} \mathop \sum \limits_{{j} = 1}^{{{d}_{x}}} {\left( {{{h}_1}\left( {1 - {{h}_1}} \right).{{w}_{{ij}}}} \right)^2} = \mathop \sum \limits_{{t} = 1}^{{{d}_{h}}} {\left( {{{h}_{i}}\left( {1 - {{h}_{i}}} \right)} \right)^2}.\mathop \sum \limits_{{j} = 1}^{{{d}_{x}}} {W}_{{ij}}^2$$

Based on these computed norm values, the reconstruction cost of proposed feature learning and steganalysis can be done accurately. The computation complexity of the proposed research method is *O* (*d_x_* × *d_h_*).

## Results and Discussion

The Dataset is comprised of over 50,000 images and out of which 10,000 images are taken for training. Over 2,500 images are taken for testing and tested thoroughly for noise removal. The authors have taken images of resolution 256 × 256 for proper quality and testing.

The proposed research methodology namely Non-Gaussian Noise Aware Auto Encoder Deep Neural Network (NGN-AEDNN) is implemented on the Matlab simulation environment and the results attained are compared with the existing methodology namely Convolutional Auto Encoder Deep Learning Framework (CAE-DLF). The illustrations are presented in the graphical representation.

To evaluate the performance of the proposed method, this work uses the following metrics for performance assessment of the NGN-AEDNN method. This work applied the proposed method on images with and without hidden information and compares the performance of the proposed compression method with other compression methods. The overall research of this work is evaluated in terms of performance measures namely

- Accuracy
- Precision
- Recall
- F-Measure

These performance measures are used to evaluate the improvement of the proposed methodology NGN-AEDNN for the prediction of steganalysis whereas the existing system used Convolutional Auto Encoder Deep Learning Framework (CAE-DLF) for prediction. A detailed explanation of numerical evaluation of the proposed and existing research methodologies are given in the following figure.

**Figure 2:**
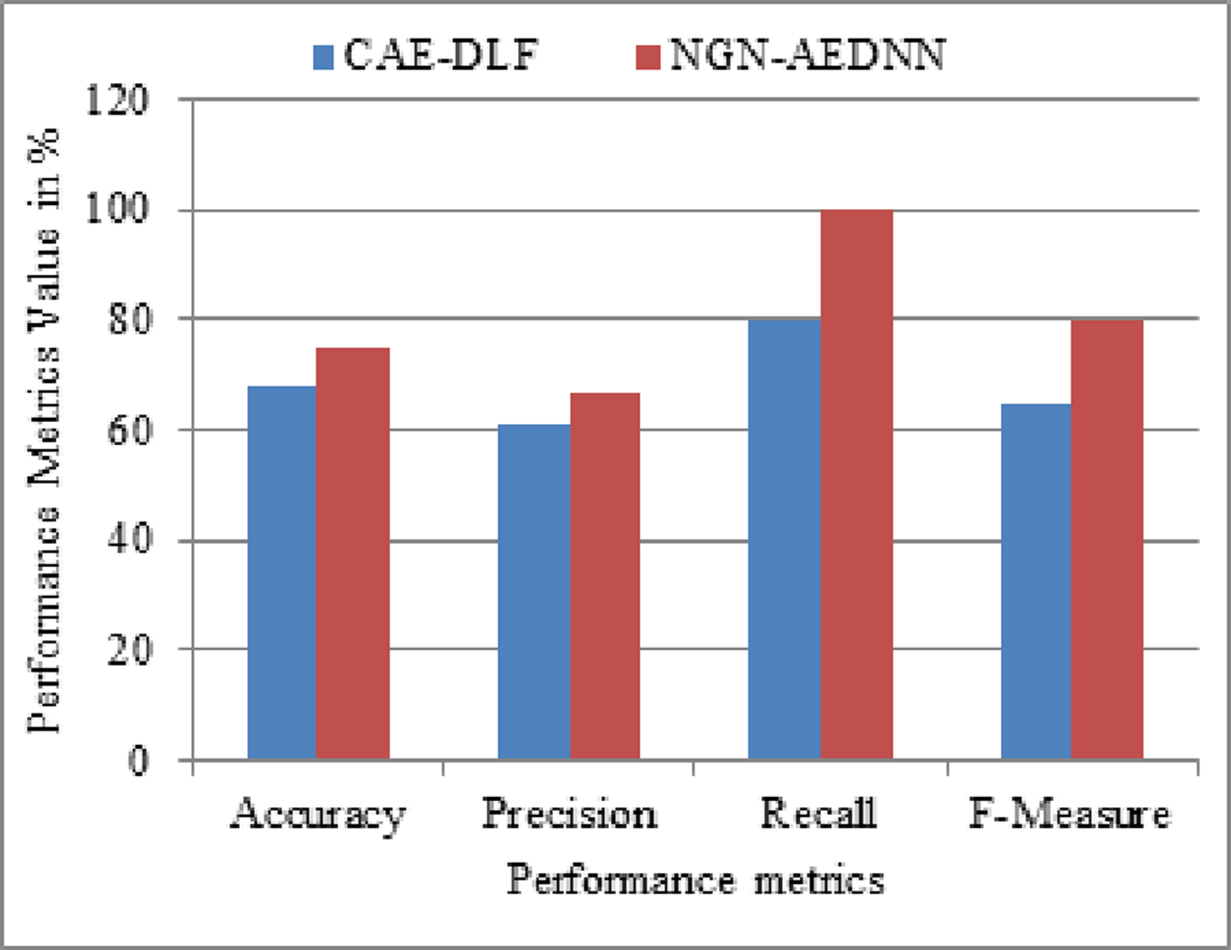
Comparison evaluation graph.

In the Fig. 2, the proposed and existing research method comparison has been done in terms of existing and proposed research methodologies. From this comparison evaluation, it can be proved that the proposed method leads to provide a better outcome than the existing research method.

## Conclusions

This is resolved in the proposed research technique by introducing the novel research method called Non-Gaussian Noise Aware Auto Encoder Deep Neural Network (NGN-AEDNN). This technique gives a more flexible way for steganalysis where the multiple features present in the environment may lead to an inaccurate prediction rate. Here, learning accuracy is improved by introducing noise removal techniques before performing the learning task. Non-Gaussian Noise Removal technique is utilized to remove the noises before learning. And also Gaussian noise removal is applied at every iteration of the neural network to adjust the error rate without the involvement of noisy features. The proposed research ensures that the optimal detection of hidden information by using the accurate learning task. The work was carried out in a Matlab simulation environment and the results are proved that the proposed NGN-AEDNN produces accurate steganalysis with reduced computational overhead when compared with the existing methods.

## Supplemental Information

10.7717/peerj-cs.356/supp-1 Supplemental Information 1 Raw trainlabel.

10.7717/peerj-cs.356/supp-2 Supplemental Information 2 Raw worldtittle.

10.7717/peerj-cs.356/supp-3 Supplemental Information 3 Raw Train feature.

## Additional Information and Declarations

### Data Availability

The following information was supplied regarding data availability: Raw data and MATLAB code are available in the Supplemental Files.
